# Efficacy and safety of selinexor-based regimens as first-line treatments for elderly patients with diffuse large B-cell lymphoma: a real-world study

**DOI:** 10.1186/s12885-025-14295-6

**Published:** 2025-05-15

**Authors:** Jing Li, Jingjing Ge, Tingting Chen, Jianghua Cao, Xiaohua He, Shulan Wang, Hang Yang, Yu Wang, Peng Sun, Jiajia Huang, Shan Liu, Zhiming Li

**Affiliations:** 1 Department of Clinical Nutrition, State Key Laboratory of Oncology in South China, Collaborative Innovation Center for Cancer Medicine, Guangdong Provincial Clinical Research Center for Cancer, Sun Yat-Sen University Cancer Center, Guangzhou, 510060 People’s Republic of China; 2 Department of Medical Oncology, State Key Laboratory of Oncology in South China, Collaborative Innovation Center for Cancer Medicine, Guangdong Provincial Clinical Research Center for Cancer, Sun Yat-Sen University Cancer Center, No.651, Dongfeng Road East, Guangzhou, 510060 People’s Republic of China; 3 Department of Hematology, First Affiliated Hospital of Southern, Shenzhen People‘s Hospital, Second Clinical Medical College of Jinan University, University of Science and Technology, Shenzhen, 518020 People’s Republic of China; 4 Department of Lymphoma, Guangdong Academy of Medical Sciences, Guangdong Provincial People‘s Hospital, Guangdong, People’s Republic of China

**Keywords:** Diffuse large B-cell lymphoma, Elderly patients, Selinexor, Efficacy, Safety

## Abstract

**Purpose:**

This study aimed to evaluate the efficacy and safety of selinexor-based regimens as first-line treatments for elderly patients with diffuse large B-cell lymphoma (DLBCL).

**Methods:**

A retrospective analysis of 16 elderly patients with DLBCL who received selinexor-based regimens as first-line treatments at Sun Yat-sen University Cancer Center from November 2021 to September 2023 was conducted. The primary endpoint was the objective response rate (ORR), while the secondary endpoints included progression-free survival (PFS), duration of response (DOR), and safety.

**Results:**

Among the 16 elderly patients, 7 were male (43.8%), and 9 were female (56.2%). The median age was 70.5 years (range, 60–80). The ORR was 93.8%, and 13 patients (81.3%) achieved a complete response (CR), 2 patients (12.5%) achieved a partial response (PR) and 1 patient had progressive disease (PD). It is noteworthy that all 5 patients who received chemotherapy-free regimens achieved CR. The median follow-up was 8.5 months (range, 2.7–22.9). The median PFS was not reached, and the 1-year PFS rate was 79.6%. A total of 81.3% of the patients maintained a response for at least 6 months, and 25% maintained a response for at least 12 months. All 3 patients aged ≥ 75 years achieved CR (100%). Haematologic AEs, including leukopenia (*n* = 15, 93.8%), neutropenia (*n* = 13, 81.3%), anaemia (*n* = 8, 50.0%) and thrombocytopenia (*n* = 4, 25.0%), were common. The most common nonhaematologic AEs were nausea and vomiting (*n* = 6, 37.5%), fatigue (*n* = 5, 31.3%) and decreased appetite (*n* = 5, 31.3%), most of which were limited in severity to grades 1 or 2 and improved with standard supportive care.

**Conclusions:**

In the real world, selinexor-based regimens demonstrate good efficacy and controllable safety as first-line treatments for elderly patients with DLBCL.

**Supplementary Information:**

The online version contains supplementary material available at 10.1186/s12885-025-14295-6.

## Introduction

Diffuse large B-cell lymphoma (DLBCL) is the most common type of non-Hodgkin lymphoma (NHL) and is aggressive and heterogeneous in nature [1]. The incidence of DLBCL increases with age, but the 5-year relative survival rate decreases with age [2]. Being over 60 years at diagnosis is an important risk factor for a high International Prognostic Index (IPI) score, which indicates poor clinical outcomes in patients who receive standard treatment. For decades, R-CHOP (rituximab plus cyclophosphamide, doxorubicin, vincristine, and prednisone) immunochemotherapy has been the first-line standard of care for DLBCL patients, curing 60% to 70% of them [3–6]. However, elderly patients generally have a lower physical fitness level, reduced tolerance to chemotherapy, and more adverse prognostic factors with a greater complexity of molecular characteristics [7]. Therefore, elderly patients tend to have lower remission rates and poorer survival status [8]. The NCCN guidelines recommend the R-miniCHOP regimen as one of the standard treatment options for elderly and frail patients, as this regimen has a two-year PFS rate of 47% and an OS rate of 59% [9]. These findings have also been confirmed in real-world studies, yet the efficacy still needs improved. Moreover, among geriatric patients with diffuse large B-cell lymphoma (DLBCL) who meet the criteria for frailty and have contraindications to R-miniCHOP due to compromised organ function, non-chemotherapeutic regimens incorporating novel targeted agents have emerged as guideline-recommended treatment alternatives. Thus, it is crucial to emphasize personalized treatment based on the individual health status and organ reserve of elderly patients.

Nuclear export protein 1 (XPO1) is a key nucleoplasmic transporter protein in cells that binds proteins, many of which are tumour suppressor proteins, or RNAs containing hydrophobic nuclear export signals (NESs) [10]. Excessive nuclear export is an important factor in cancer development and is associated with chemotherapy resistance [11]. Selinexor is the world's first approved oral selective nuclear export protein inhibitor and was approved by the U.S. Food and Drug Administration (FDA) for the treatment of recurrent or refractory (R/R) DLBCL and R/R multiple myeloma (MM) [12, 13]. Selinexor selectively targets XPO1 and contributes to the intranuclear storage and activation of tumour suppressor proteins and other growth-regulating proteins, downregulates the levels of multiple oncogenic proteins in the cytoplasm and induces apoptosis of tumour cells [14–16]. Several clinical trials have demonstrated the superior clinical efficacy and safety of selinexor as a single agent or as a combination therapy for the treatment of DLBCL [13, 17]. However, the efficacy and safety of selinexor have not been explored in elderly patients. Therefore, we retrospectively collected real-world, single-centre cases to analyse the efficacy and safety of selinexor-containing regimens for the treatment of elderly patients with DLBCL.

## Materials and methods

### Patients and treatments

The clinical data of 16 elderly patients with DLBCL who were treated at Sun Yat-sen University Cancer Center from November 2021 to September 2023 and who completed at least 2 cycles of first-line treatment with selinexor-containing regimens were retrospectively collected. All patients were aged 60 years or older, had a life expectancy of at least 3 months and included high-risk populations such as those with stage III-IV disease and those with an IPI score ≥ 3, extranodal involvement ≥ 2, double expression lymphoma (DEL) and double- or triple-hit lymphoma (DHL/THL). The diagnosis was consistent with the revised classification criteria for lymphoid tissue tumours by the World Health Organization (WHO) in 2016 [18]. All patients received selinexor combined with chemotherapy (R-CHOP or R-miniCHOP) or chemotherapy-free regimens, including rituximab and lenalidomide (R2) and rituximab and orelabrutinib (R-O). The dose of selinexor was 40 mg orally per week (days 1, 8, and 15 of the 28-day cycle). As human participants were involved, this study was reviewed and approved by the ethics committee of Sun Yat-sen University Cancer Center. This study’s protocol complied with the Declaration of Helsinki, and the patients provided written informed consent prior to their participation in the study.

### Evaluation and definition

Efficacy was assessed according to the revised 2014 Lugano criteria for the assessment of treatment response in lymphoma [19], which included complete response (CR), partial response (PR), stable disease (SD) and progressive disease (PD). Positron emission tomography/computed tomography (PET/CT) was performed for systemic disease evaluation every 2 treatment cycles, and an independent oncologist reviewed the clinical data and confirmed the best response, duration, and disease progression. The primary endpoint of the study was the objective response rate (ORR), which was defined as the proportion of patients with an optimal response of CR or PR. One secondary endpoint was progression-free survival (PFS), which is defined as the duration of time from the initiation of selinexor-containing regimens until progression, death due to any cause or last follow-up. Another secondary endpoint was duration of response (DOR), which is defined as the time from the first occurrence of CR or PR until disease progression was objectively documented. Adverse events (AEs) were graded according to the National Cancer Institute Common Terminology Criteria for Adverse Events (CTCAE) version 5.0 and were summarized for all patients who received treatment.

### Statistical analysis

SPSS 26.0 and GraphPad Prism 8.0 were used for statistical analysis. Descriptive statistics were used to represent the clinical baseline characteristics and treatment status of the patients. Continuous variables are presented as medians (ranges), and categorical variables are presented as frequencies and percentages. Survival curves were generated using the Kaplan‒Meier method.

## Results

### Patient characteristics

Sixteen elderly patients with newly treated DLBCL were included in this study, including 7 males (43.8%) and 9 females (56.2%). The median age of patients throughout the course of treatment was 70.5 years (range 60–80 years), and 3 patients (18.8%) were ≥ 75 years. Eight patients (50.0%) had Ann Arbor stage III-IV disease, and 7 patients (43.8%) had an IPI score ≥ 3. Fourteen patients (87.5%) were diagnosed with the GCB subtype, 4 patients (25.0%) had DEL, and 2 patients (12.5%) had DHL or THL. Of the 16 patients, 6 (37.5%) had elevated LDH, 6 patients (37.5%) were found to have extranodal involvement ≥ 2 and 12 patients (75.0%) had a Ki-67 index ≥ 80%. The detailed clinical information of the patients with DLBCL is shown in Table 1.

**Table 1:** Characteristics of the patients with DLBCL

Characteristics	N (%)
**Age (years)**	
Median (range)	70.5 (60–80)
≥ 75 year	3 (18.8)
**Sex**	
Male	7 (43.8)
Female	9 (56.2)
**Ann Arbor staging**	
I-II	8 (50.0)
III-IV	8 (50.0)
**IPI score**	
0–2	9 (56.2)
≥ 3	7 (43.8)
**ECOG Performance Status**	
0–1	13 (81.3)
≥ 2	3 (18.7)
**LDH (u/L) > UNL**	
Yes	6 (37.5)
No	10 (62.5)
**β2-MG (ng/L) > UNL**	
Yes	4 (25.0)
No	11 (68.8)
Unknown	1 (6.2)
**DLBCL subtype**	
GCB	14 (87.5)
Non-GCB	2 (12.5)
**Extranodal involvement**	
≥ 2	6 (37.5)
< 2	10 (62.5)
**MYC expression**	
≥ 40%	4 (25.0)
< 40%	12 (75.0)
**Ki-67**	
≥ 80%	12 (75.0)
< 80%	4 (25.0)
**Double or triple hit lymphoma (DHL/THL)**	2 (12.5)
**Double expressor lymphoma (DEL)**	4 (25.0)
**Treatment regimens**	
Selinexor plus R-CHOP	6 (37.5)
Selinexor plus R-miniCHOP	5 (31.3)
Selinexor plus R2	4 (25.0)
Selinexor plus R-O	1 (6.2)
**Treatment cycles, Median (range)**	6 (3–6)
**Action because of adverse reaction**	
Dose reduction	1 (6.2)
Discontinuation	1 (6.2)

### Efficacy and survival

**Fig. 1:**
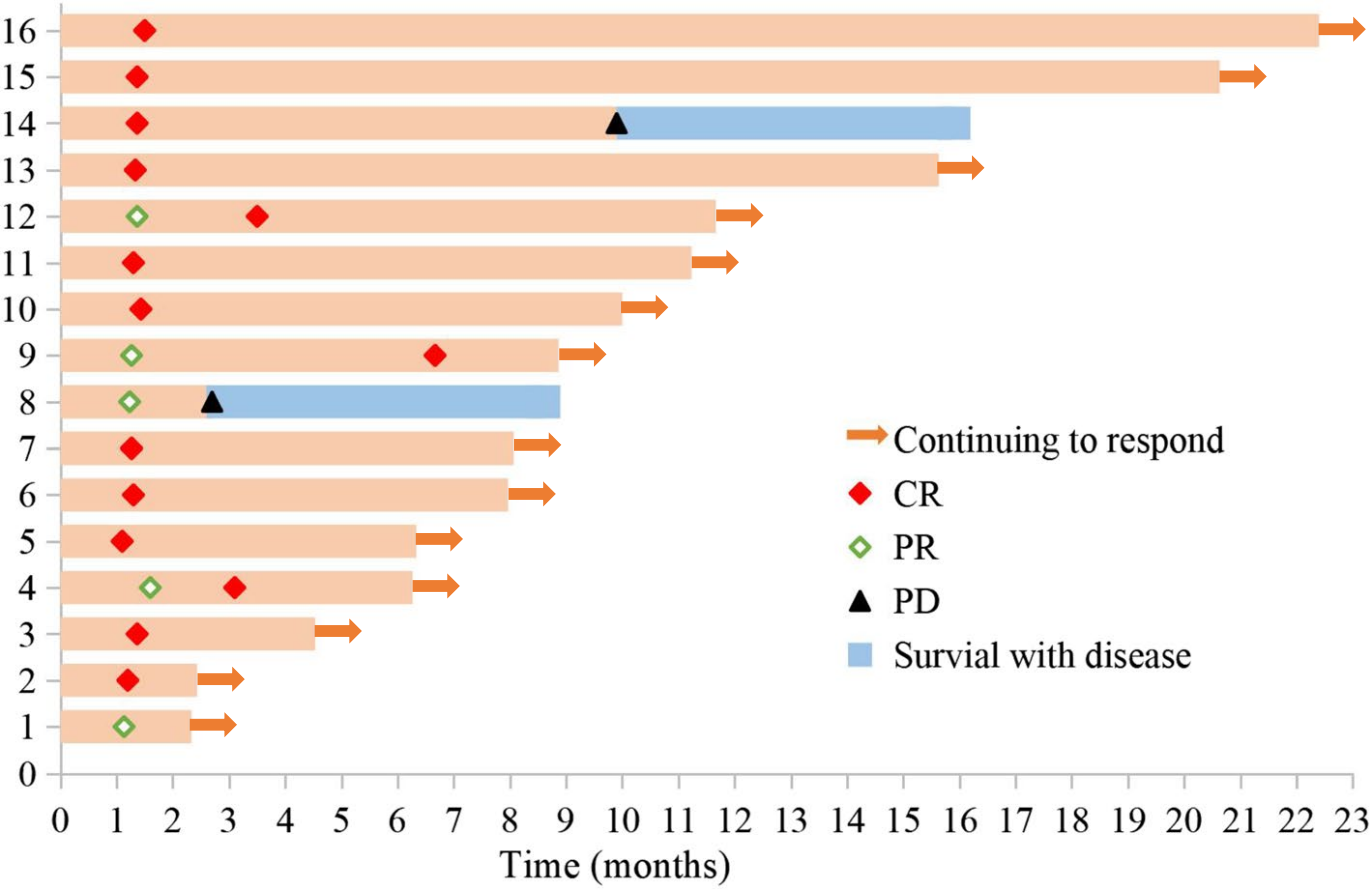
Swimmers plot illustrating a summary of clinical courses for the 16 patients

**Fig. 2:**
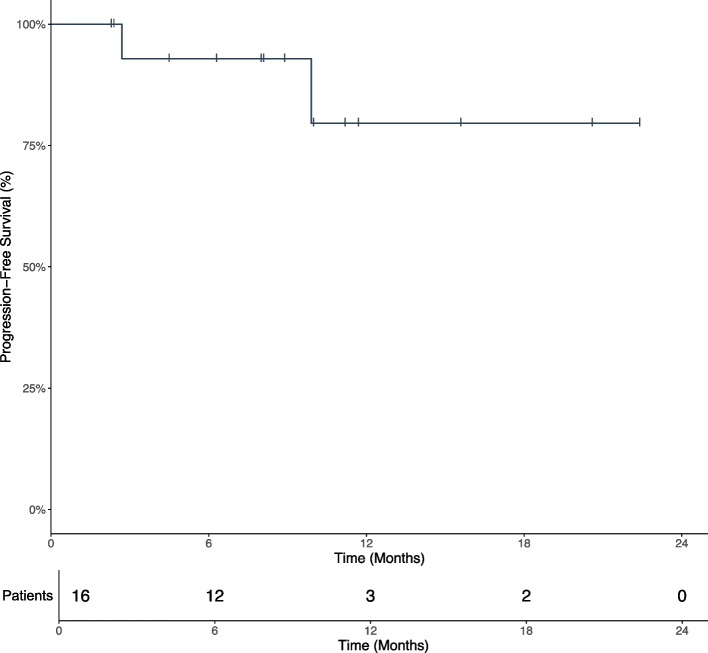
PFS for the 16 patients

Eleven patients (68.8%) received selinexor combined with chemotherapy, including 6 cases of selinexor plus R-CHOP and 5 cases of selinexor plus R-miniCHOP. These regimens were selected based on a comprehensive geriatric assessment conducted by attending physicians, which included evaluations of age, overall health status, organ function, and frailty status. Five patients (31.2%) received selinexor combined with chemotherapy-free regimens, determined by attending physicians'assessments of comorbidities and overall health status or by patient preference. Among them, 4 patients who received selinexor plus R2 and 1 patient who received selinexor plus R-O. Twelve patients completed all 6 cycles of treatment, and at the time of this writing, 4 patients were still receiving therapy, all of whom could still be evaluated for efficacy. The ORR was 93.8%, and 13 patients (81.3%) achieved CR, 2 patients (12.5%) achieved PR and 1 patient discontinued treatment because of PD. The median follow-up time was 8.5 months (range, 2.7–22.9). During this period, one advanced high-risk patient with multiple systemic involvement and bulky disease achieved CR after 6 cycles of treatment, but experienced disease progression after 8 months of remission. Second-line treatment with different regimens failed to achieve CR and the patient was undergoing third-line treatment. A swimmers plot demonstrated the response to treatment in 16 patients (Fig. 1). The median PFS was not reached, and the 1-year PFS rate was 79.6% (Fig. 2). The median DOR was 8.8 months (range, 2.3–22.4), 81.3% of the patients maintained a response for at least 6 months, and 25% of the patients maintained a response for at least 12 months.

Subgroup analyses revealed that 3 patients aged ≥ 75 years achieved CR (100%). The ORR was 90.9% in 11 patients who received selinexor combined with chemotherapy, and of these, 8 patients achieved CR (72.7%). All 5 patients obtaining CR (100%) were treated with chemotherapy-free regimens. Among the 14 patients with the GCB subtype, the ORR was 100%, with 12 patients achieving CR (85.7%) and 2 patients achieving PR (14.3%). The ORR was 87.5% in 8 patients with stage III-IV disease, of whom 5 patients achieved CR (62.5%), 2 patients achieved PR (25.0%), and 1 patient experienced PD (12.5%). Five of the six patients with extranodal involvement ≥ 2 were in remission and had an ORR of 83.3%. The ORR and CR in 4 patients with DEL and 2 patients with DHL were 100% and 50.0%, respectively (Table 2).

**Table 2:** Responses in evaluable patients

	**ORR**	**CR**	**PR**	**PD**
All patients	15/16 (93.8)	13 (81.3)	2 (12.5)	1 (6.2)
≥ 75 year	3/3 (100)	3/3 (100)	-	-
Selinexor combined with Chemotherapy	10/11 (90.9)	8 (72.7)	2(18.2)	1 (9.1)
Selinexor combined with Chemotherapy-free	5/5 (100)	5 (100)	-	-
GCB subtype	14/14 (100)	12 (85.7)	2 (14.3)	-
IPI ≥ 3	6/7 (85.7)	4 (57.1)	2 (28.6)	1 (14.3)
Ann Arbor staging (III-IV)	7/8 (87.5)	5 (62.5)	2 (25.0)	1 (12.5)
Extranodal involvement ≥ 2	5/6 (83.3)	4 (66.6)	1 (16.7)	1 (16.7)
Ki-67 ≥ 80	11/12 (91.7)	9 (75.0)	2 (16.7)	1 (8.3)
DHL/THL	2/2 (100)	1 (50.0)	1 (50.0)	-
DEL	4/4 (100)	2 (50.0)	2 (50.0)	-

### Safety

Sixteen patients experienced at least one treatment-related AE (Table 3). Haematologic AEs of any grade were common and included leukopenia (n = 15, 93.8%), neutropenia (n = 13, 81.3%), anaemia (n = 8, 50.0%) and thrombocytopenia (n = 4, 25.0%). Grade 3/4 haematologic AEs were uncommon, as only 5 patients (31.3%), 3 patients (18.8%) and 1 (6.2%) patient experienced neutropenia, leukopenia and thrombocytopenia, respectively. The most common nonhaematologic AEs were nausea and vomiting (n = 6, 37.5%), fatigue (n = 5, 31.3%) and decreased appetite (n = 5, 31.3%), most of which were limited in severity to grades 1 or 2 and improved with standard supportive care. One patient (6.2%) developed pulmonary infection after 2 cycles of selinexor plus R-miniCHOP treatment, which improved after intravenous antibiotics, and after which the dose of selinexor was adjusted to 20 mg at the start of the third cycle. Another patient developed acute heart failure after selinexor plus R-CHOP and recovered after symptomatic treatment. Although the incidence of leukopenia and neutropenia was high, no fatal outcomes were observed because febrile neutropenia usually resolved after standard growth factor and antibiotic treatment.

**Table 3:** Treatment-related adverse events

Adverse Events	All	Grade 1–2	Grade 3–4
Leukopenia	15 (93.8)	12 (75.0)	3 (18.8)
Neutropenia	13 (81.3)	8 (50.0)	5 (31.3)
Anaemia	8 (50.0)	8 (50.0)	-
Thrombocytopenia	4 (25.0)	3 (18.8)	1 (6.2)
Fatigue	5 (31.3)	5 (31.3)	-
Nausea/vomiting	6 (37.5)	5 (31.3)	1 (6.2)
Decreased appetite	5 (31.3)	5 (31.3)	-
Headache	1 (6.2)	1 (6.2)	-
Pneumonia	1 (6.2)	-	1 (6.2)
Oedema peripheral	1 (6.2)	1 (6.2)	-
Fever	4 (25.0)	4 (25.0)	-
Numbness	2 (12.5)	2 (12.5)	-
Cardiac failure	1 (6.2)	1 (6.2)	-

## Discussion

To our knowledge, this is the first retrospective study that has evaluated the efficacy and safety of selinexor-containing regimens for the treatment of previously untreated elderly DLBCL patients. This study demonstrated encouraging efficacy of selinexor-based regimens, with an ORR of 93.8% (CR rate of 81.3%), a median follow-up of 8.5 months, and a CR rate of 100% for patients ≥ 75 years of age. However, further follow-up is needed to obtain survival data in elderly patients. This study demonstrated that the addition of selinexor to the R-CHOP or R-miniCHOP regimens improved efficacy with a manageable safety profile in elderly DLBCL patients who could tolerate immunochemotherapy. Moreover, Selinexor combined with chemo-free regimens demonstrated efficacy and safety in elderly DLBCL patients with significant comorbidities that were anticipated to compromise chemotherapy tolerance (as assessed by the treating physician) or based on patient preference.

Given the increase in ageing populations worldwide, the treatment and overall management of elderly DLBCL patients have gradually become prominent clinical issues. Compared with younger patients, DLBCL patients aged > 60 years are characterized by advanced stage disease, poor Eastern Cooperative Oncology Group (ECOG) status, elevated LDH, and high rates of extranodal involvement and have significantly different prognoses [20]. The age inclusion criterion (≥ 60 years) in this study is based on the 2024 CSCO Guidelines'recommendation for geriatric assessment in DLBCL patients aged ≥ 60. Although some patients are under 70, their high-risk features (such as IPI ≥ 3, high Ki-67) and individualized treatment needs (like reduced-dose chemotherapy or chemo-free regimens) greatly overlap with the typical elderly population. A national, population-based study from the Netherlands reported relative survival data for patients with DLBCL of different ages and stages [21]. The five-year relative survival rates for the 18–64, 65–74, and > 75 year age groups in patients with stage I disease were 96%, 84%, and 67%, respectively, whereas they were 75%, 60%, and 46%, respectively, in patients with stages II–IV disease. Notably, most deaths in elderly patients are due to lymphoma progression, which clearly necessitates improved treatment efficacy. Elderly patients with DLBCL often have multiple comorbidities, such as cardiovascular disease, renal disease, diabetes and other chronic diseases [22]. Considering organ function and adverse drug events, the use of standard therapeutic regimens in the real world is typically limited by dosage, which results in diminished efficacy. In addition, a subset of patients may be at risk of death due to treatment-related adverse events, and thus they may require admission to the intensive care unit (ICU). Therefore, the balance of efficacy and safety should be considered in the overall management of elderly patients with DLBCL.

The approval of selinexor for use in DLBCL was based on the SADAL phase II study [13, 23], which enrolled 134 patients with R/R DLBCL who had received ≥ 2 lines of prior therapy. All patients were treated with oral selinexor monotherapy, which resulted in an ORR of 29.1% and a DOR of 9.3 months. The median overall survival (OS) was 9 months, and the patients who achieved CR and PR also achieved a median OS of 29.7 months. Notably, the median age of the patients in this study was 67 years, 44.8% of the patients were ≥ 70 years, and their median OS was 7.8 months, which suggests that selinexor monotherapy was effective and safe for R/R elderly DLBCL patients.

Several clinical trials have evaluated the efficacy of new drugs combined with the R-CHOP regimen in the first-line treatment of DLBCL. The POLARIX study demonstrated that the addition of polatuzumab-vedotin to first-line immunochemotherapy improved PFS [24]. In contrast, some studies have shown that the addition of drugs to the treatment regimens of different subgroups of DLBCL patients could increase their efficacy or survival benefits [25, 26]; for example, the venetoclax plus R-CHOP regimen improved the outcomes of patients with Bcl-2-positive DLBCL [27]. In the PHOENIX study [26], first-line ibrutinib in combination with R-CHOP showed a survival benefit in younger patients (aged < 60 years), but in patients aged 60 years or older, the regimen was associated with increased toxicity, which affected the therapeutic benefit in those patients. While the standard R-CHOP therapy is a well-established and effective treatment for DLBCL patients aged 60 to 80 years, as reported in the literature [28], our research primarily concentrated on evaluating the efficacy of selinexor-based regimens. This study is the first to report that the ORR of a selinexor-based regimen as a first-line treatment for elderly DLBCL patients was 93.8%. Among these patients, 11 received selinexor plus R-CHOP or R-miniCHOP, with an ORR and a CR rate of 90.9% and 72.7%, respectively. Similar to what was observed in our study, previously untreated NHL patients treated with selinexor in combination with R-CHOP had an ORR of 100% (10/10) and a CR rate of 90% [29]. Selinexor significantly increased CD20 expression on the surface of B-NHL cells, inhibited the proliferation of double-hit (Bcl-2 and C-Myc mutation-positive) lymphoma cell lines, and exerted a remarkable synergistic effect when combined with CHOP [29]. The efficacy and safety of the selinexor plus R-CHOP/R-miniCHOP regimen, which has a novel synergistic mechanism of action, were further demonstrated.

The Smart Start study explored the efficacy of the RLI regimen (rituximab, lenalidomide and ibrutinib) with the sequential addition of chemotherapy in newly diagnosed nongerminal centre B-cell-like DLBCL patients. The patients had a median age of 63.5 years (range, 29–83) and of all the patients, 28% were aged ≥ 70 years. After two cycles of RLI alone, the ORR was 86.2%, and the complete response rate reached 94.5% after the completion of RLI chemotherapy [30]. Zhao's team explored the efficacy and safety of the ibrutinib, rituximab and lenalidomide (IR2) regimen in elderly DLBCL patients who were too unfit/frail for standard chemotherapy [31]. According to the geriatric assessment (GA) [32], frail patients have poorer survival and are less likely to receive curative therapy [33]. The median age of the patients was 80 years (range 76–92). The CR rate of 30 patients at the end of induction therapy was 56.7%, the ORR was 66.7%, and the median follow-up duration was 27.6 months, with a 2-year PFS rate of 53.3% and a 2-year OS rate of 66.7%. Similarly, the phase II FIL_ReRi trial explored lenalidomide plus rituximab (R2) in frail untreated elderly DLBCL patients (median age: 83 years; range: 70–91), demonstrating the feasibility of immunomodulatory regimens in this vulnerable population [34]. At the end of induction (EOI), 27.7% of patients achieved CR, and the ORR was 50.8%. The 2-year PFS was 40.5%, and the median PFS was 14.0 months. These results support the finding that the chemotherapy-free regimen is safe and efficacious in elderly patients. In addition, preclinical and clinical data have shown that selinexor has synergistic effects with chemotherapy, BTK inhibitors and PD-1 or PD-L1 monoclonal antibodies [18, 20, 35, 36] and provides clinical benefits to patients. In this study, five patients who received the selinexor plus chemotherapy-free combination regimen refused chemotherapy due to their comorbidities, overall health status, or personal preferences. Four of these elderly patients were treated with selinexor plus R2 and had a CR rate of 100%. One DHL patient who achieved CR, which was sustained after two cycles, was treated with a combination of selinexor, rituximab and orelabrutinib. All patients achieved CR after treatment, possibly because 80% (4/5) of patients had stage I-II disease with less extranodal involvement and a lower IPI score. Selinexor in combination with chemotherapy-free regimens is a novel option for the treatment of elderly patients with DLBCL.

Myelosuppressive haematological AEs, including leukopenia, anaemia, neutropenia and thrombocytopenia, are common complications due to the relative lack of bone marrow function in elderly patients. No treatment discontinuation due to toxicity occurred in the 16 patients in our study, compared with the 21.5% in the FIL_ReRi study [34] and the 2 patients in the IR2 study who terminated treatment due to adverse reactions [31] (Supplementary Table 1). Supportive therapy, including prehormonal therapy and the use of prophylactic granulocyte colony-stimulating factor (G-CSF), is important for the prevention of AEs in elderly patients [37]. Elderly patients with comorbid underlying diseases should be closely monitored for organ function and specialized symptomatic management.

The main limitations of the current study were its retrospective design and small sample size. Due to the short period of time since selinexor was approved, the follow-up time in this study was insufficient, and thus long-term follow-up of the patients will be performed. In addition, the sample size of this real-world study was small, but AEs are more likely to be observed in elderly patients, especially in subjects who would typically be excluded from clinical trials.

## Conclusions

In conclusion, the current treatment of elderly patients with DLBCL remains a formidable challenge for physicians. The choice of treatment should be individualized and should consider a balance of efficacy and toxicity. Considering the results of this real-world study, selinexor-based regimens are promising regimens with less toxicity that can be used in previously untreated elderly DLBCL patients. However, larger sample sizes and long-term follow-up are needed to further evaluate the efficacy of this treatment approach.

## Supplementary Information

Supplementary Material 1.

## Data Availability

No datasets were generated or analysed during the current study.
