# Supplementary material for: Efficacy and safety of selinexor-based regimens as first-line treatments for elderly patients with diffuse large B-cell lymphoma: a real-world study
Source: BMC Cancer. 2025 May 15;25:878. doi: 10.1186/s12885-025-14295-6 (PMC12079892; doi:10.1186/s12885-025-14295-6)
Supplement: Supplementary file 1 — Supplementary Material 1. [file 12885_2025_14295_MOESM1_ESM.docx]

**Supplementary Table 1. Comparative Analysis of Outcomes and Toxicities in Elderly DLBCL Studies**

| **Study** | **Patient Characteristics** | **Clinical Outcomes** | **Treatment-related Toxicities** |
| --- | --- | --- | --- |
| This Study (Selinexor-based regimens) | - Number of patients: 16  - Median age: 70.5 years (range 60-80)  - 18.8% aged ≥75 years | - ORR: 93.8%  - CR rate: 81.3% (13 patients)  - Median PFS: Not reached  - 1-year PFS rate: 79.6%  - Median follow-up: 8.5 months (range 2.7-22.9) | - Haematologic AEs: - Any grade: leukopenia (93.8%), neutropenia (81.3%), anaemia (50.0%), thrombocytopenia (25.0%) - Non-Haematologic AEs: - Nausea and vomiting (37.5%), fatigue (31.3%), decreased appetite (31.3%)  - No treatment discontinuation due to toxicity |
| Smart Start study (RLI regimen)^30^ | - Number of patients: 60  - Median age: 63.5 years (range 29-83) - 28% aged≥70 years | - ORR after 2 cycles of RLI alone: 86.2% - CR rate after RLI chemotherapy completion: 94.5%  - 2-year progression-free survival: 91.3%  - 2-year overall survival: 96.6%  - Median follow-up: 31 months | - Haematologic AEs: - Thrombocytopenia grade 2-4 (58.0%), neutropenia grade 3-4 (53.0%), anaemia grade 2-4 (53.0%), febrile neutropenia (37.0%) - Non-Haematologic AEs: - Nausea (85.0%) , peripheral sensory neuropathy (83.0%) , diarrhea (78.0%), mucositis (75.0%), rash (53.0%) , dyspnea(43.0% ) |
| Zhao's team (IR2 regimen)^31^ | - Number of patients: 30  - Median age: 80 years (range 76-92) | - CR rate at end of induction: 56.7% - ORR: 66.7% - 2-year PFS: 53.3% - 2-year OS: 66.7% - Median follow-up: 27.6 months | - Haematologic AEs: - Any grade: neutropenia (43.0%), anaemia (30.0%), thrombocytopenia (27.0%) - Non-Haematologic AEs: - Pulmonary infection (23.0%), ALT or AST elevation (17.0%), atrial fibrillation (10.0%), hyperuricaemia (23.0%)  - 2 patients terminated treatment due to adverse reactions |
| FIL_ReRi trial (R2 regimen)^34^ | - Number of patients: 65  - Median age: 83 years (range 70-91) | - CR at end of induction: 27.7% - ORR: 50.8% - 2-year PFS: 40.5% - Median PFS: 14.0 months | - Haematologic AEs: - Grade ≥3: neutropenia (46.1%), anaemia (3.1%), thrombocytopenia (9.3%)  - Non-Haematologic AEs:  - Grade ≥3: respiratory/thoracic and mediastinal disorders (14.0%), general disorders/administration site conditions (14.0%), skin and subcutaneous tissue disorders (14.0%), cardiac disorders (12.0%), vascular disorders (12.0%), infections (8.0%)  - 21.5% treatment discontinuation due to toxicity |
